# Large Language Models for Clinical Trial Protocol Assessments

**DOI:** 10.1002/cpt.70096

**Published:** 2025-10-21

**Authors:** Euibeom Shin, Amruta Gajanan Bhat, Murali Ramanathan

**Affiliations:** ^1^ Artificial Intelligence & Clinical Pharmacology Laboratory, Department of Pharmaceutical Sciences University at Buffalo, The State University of New York Buffalo New York USA

## Abstract

The purpose was to evaluate the utility of large language models (LLMs) for reviewing the statistical analysis plan (SAP) and pharmacokinetics–pharmacodynamics (PK–PD) components of clinical trial protocols. Clinical trial protocols and SAPs were obtained from clinicaltrials.gov for a testbed of 15 small‐molecule drugs, biologics, and global antibiotic and public health interventions. The GPT‐4o (ChatGPT) LLM was used to elicit study design attributes, relevant guidelines, and detailed SAP evaluations with prompts engineered to the persona of a regulatory expert. The SAP methodology was assessed against the Food and Drug Administration’s (FDA) E9 Statistical Principles for Clinical Trials guidance. The SAP evaluation outputs were assessed in post hoc analyses with ChatGPT and Grok, based on a rubric that evaluated the accuracy of primary outcome identification, the correctness of statistical methodology, compliance with the FDA E9 guidance, and clinical interpretability. PK–PD analysis plans were assessed on the accuracy of PK–PD objectives and measures and PK analysis methods. ChatGPT accurately identified the disease, intervention, and comparator groups for all trials, as well as the study sample size for 14 out of 15 trials. The most frequently cited guidelines were the FDA’s E9 guidance for SAP and the FDA Guidance for Industry: Population Pharmacokinetics for PK–PD. ChatGPT outputs of the SAP and PK–PD analysis plans were clear and organized, demonstrating a satisfactory ability to extract and summarize technical details; some limitations in contextual accuracy were observed. LLMs can be effective tools for assessing the SAP, PK–PD, and other aspects of clinical trial protocol reviews.

**Study Highlights**

**WHAT IS THE CURRENT KNOWLEDGE ON THE TOPIC?**

Large language models (LLMs) are an artificial intelligence technology with transformative potential for clinical pharmacology. LLMs are being evaluated as a productivity tool for various coding and writing tasks in clinical pharmacology; however, the utility of LLMs in guiding decision making has not been systematically assessed.

**WHAT QUESTION DID THIS STUDY ADDRESS?**

The goal was to determine whether LLMs could be leveraged as a decision‐making tool for evaluating clinical trial protocols. A testbed of 15 small‐molecule, biologics, and global trials was used to assess the performance of the LLM on key tasks, including the extraction of trial study design attributes, the identification of relevant regulatory guidelines, and the detailed evaluation of testing procedures in the SAP.

**WHAT DOES THIS STUDY ADD TO OUR KNOWLEDGE?**

ChatGPT performed well in identifying trial study design attributes, including disease, intervention, and comparator groups, as well as the study sample size and clinical trial protocol. ChatGPT also provided regulatory guidelines for statistical analysis plans and pharmacokinetics–pharmacodynamics. ChatGPT demonstrated satisfactory performance in extracting the statistical procedures and providing a detailed point‐by‐point summary and evaluation.

**HOW MIGHT THIS CHANGE CLINICAL PHARMACOLOGY AND THERAPEUTICS?**

LLMs could be useful as an aid for reviewing clinical trial protocols in high‐volume settings, such as those of regulatory and funding agencies.

Large language models (LLMs) are extremely large deep‐learning‐based artificial intelligence (AI) systems containing billions of parameters that are trained on vast amounts of data. Examples of LLMs include ChatGPT from OpenAI, the first LLM released; Gemini, Llama, Claude, Grok, and DeepSeek.^1^, ^2^, ^3^, ^4^, ^5^, ^6^, ^7^ LLMs can emulate humans in text creation and comprehension, and their development marks a significant AI advancement.^8^

LLMs have shown promise and are being cautiously evaluated for applications in clinical pharmacology, pharmacometrics, drug development, and pharmacy counseling.^9^, ^10^, ^11^ LLMs possess generative abilities and complex reasoning skills that could benefit clinical research and decision support. However, their performance in these settings has not been widely examined. This research aimed to critically evaluate ChatGPT’s effectiveness in assessing statistical analysis plans (SAPs) and population PK–PD components of clinical trial protocols.

The Good Clinical Practice (GCP) Guideline of the International Council for the Harmonisation of Technical Requirements for Pharmaceutical Products for Human Use (ICH) provides a widely accepted structured framework and a list of topics to be covered in a clinical trial protocol.^12^ Nonetheless, clinical trial protocols can be lengthy and complex, ranging from 50 to 100 pages, with some over 200 pages, and addressing about 20–40 key areas, including site selection, sample size justification, study intervention procedures, study end points, and SAPs. As a result, the manual review of clinical trial protocols requires considerable expertise and resources. Regulatory agencies, funders, and institutional review boards receive clinical trial protocols on an ongoing basis, and clinical protocol evaluations are central to their missions. The logistical challenges of the clinical trial protocol review process present risks and opportunities. We hypothesized that LLMs could help identify inconsistencies, best practices, or red flags that may not be immediately apparent to human reviewers.

Clinical trial repositories such as clinicaltrials.gov and clinicaltrialsregister.eu are useful for obtaining study design attributes for initiated, ongoing, and completed or terminated clinical trials. However, funders, regulatory agencies, and institutional boards review clinical trial protocols prior to initiation, and obtaining study design attributes using LLM can add value for statistical and PK–PD reviewers, who often focus on the relevant sections but need sufficient context on the overall clinical trial. SAPs are critical for effectively interpreting the outcomes of clinical trials. Weak statistical methodology can increase the risk of false‐positive and false‐negative findings regarding trial outcomes. Likewise, population PK–PD models play a central role in dose selection and the design of dosing regimens. Inadequate consideration of population PK–PD, however, is costly as it can lead to a poor choice of dosing regimen, failed clinical trials, and potential harm to patients.

The goal of the research was to systematically evaluate the performance of LLM for reviewing the study design attributes, SAP, and population PK–PD components of clinical trial protocols.

## METHODS

### Protocol selection

A set of 15 phase I–III clinical protocols consisting of five trials of small‐molecule drugs (NCT02450539, NCT04613518, NCT04756531, NCT05442775, and NCT04497883), five trials of large‐molecule drugs (biologics, NCT01866319, BH29884, NCT01642004, NCT01358175, and NCT02277743), and five global trials (NCT03678688, NCT02661178, NCT03167242, NCT04523831, and NCT02968849) of antibiotics and public health‐related interventions were selected as a testbed.

The protocols were manually downloaded from clinicaltrials.gov and are summarized in **Tables** **S1** **–** **S3**. All selected trials either had clinical trial protocols, including SAP or separate clinical trial protocols and SAP documents available, and the combined document set was greater than 50 pages long.

In the following, S1–S5 refers to the small‐molecule trials, B1–B5 refers to the biological trials, and G1–G5 refers to the global trials.

### Large language models

ChatGPT 4o^13^ was used in all experiments and was run at chat.openai.com on a MacBook Air computer running macOS Sequoia 15.2. Screenshots from individual ChatGPT runs were saved. Grok 3 (www.grok.com) was employed to evaluate ChatGPT outputs using a grading rubric, with the scoring criteria summarized in Table S4.^6^ ChatGPT 4o and Grok 3 are hereinafter referred to as ChatGPT and Grok, respectively.

### Evaluation of study design attributes and protocol summary

Each of the 15 protocols was provided as input, along with a standardized prompt instructing ChatGPT to extract nine study attributes from the documentation. The study attribute variables were site locations, population, disease, sample size, intervention, comparator, trial phase, primary end point, and funders.

The prompts used to obtain the study design attributes and protocol summary are summarized in **Table** **1**.

**Table 1:** Prompts used to obtain evaluations of the study design attributes and protocol summary with ChatGPT

Prompt engineering
You are an expert at the United States Food and Drug Administration (FDA) with 20 years of regulatory experience reviewing clinical trial protocols.
Provide the following information from the clinical trial protocol, which is attached.
Eliciting study design attributes and protocol summary
Site locations—Record the total number of sites followed by countries in brackets separated by semicolons, for example, 23 (Cameroon; Chad; Nigeria)
Population—Enter a brief description of the main characteristics of the population, which may include age, location and health descriptors (e.g., Children in Africa with uncomplicated severe anemia). Record as simply as possible in line with population description
Disease—Record the disease under study in simple terms (e.g., uncomplicated severe anemia). Do not include specific eligibility criteria. If the trial recruited a healthy population, for example, to study prophylaxis or vaccines and record the disease(s) being prevented
Sample size—Enter the total number of participants who underwent randomization. For cluster randomized trials, enter the number of clusters randomized followed by the number of participants separated by a semicolon (e.g., for a cluster trial with 20 clusters and a total of 1000 participants, enter “20; 1000”)
Intervention(s)—Record short‐form names with dose where relevant for the primary intervention(s) under study, separated with semicolons if required (e.g., Prednisone 30 mg and 60 mg). If it is unclear which arm(s) is/are the intervention(s), check the title and abstract (background and methods) for quick reference of which arm(s) is/are being tested as the primary interest as the intervention(s). Established/standard care arms and low‐intervention or placebo groups will almost always be comparators rather than interventions. If still unclear, use judgment and flag uncertainty in the Flags field at the end of the form
Comparator(s)—Record short‐form names for the groups used for comparison with active comparator name in brackets and separated with semicolons if required (e.g., Active (methotrexate); Placebo)
Trial phase—Record as a single number (e.g., 2) or a combination of phases separated by a forward slash (e.g., 1/2a). If phase is not mentioned in the publication, check the trial registry record, if available, for phase information. If no phase information can be found, apply the clinicaltrials.gov phase definitions (https://clinicaltrials.gov/studybasics/glossary)
Primary end point—Record the primary end point specified in the paper. If a list of outcomes is given but no primary outcome is specified, record it as NR
Funders—Record each funder separated with semicolons if required

#### Evaluation criteria

The accuracy of ChatGPT’s responses was assessed by identifying inconsistencies with the original clinical trial protocol document and the trial summary on clinicaltrials.gov. Accuracy evaluations were done by the first author, E.S.

The LLM output was graded using the following criteria: Accurate 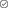: LLM results match both the protocol and clinicaltrials.gov sources; Incorrect 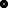: LLM result is inaccurate; Partially Incorrect 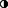: The response is missing information; Missing 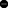: Protocol is missing Information.

### Evaluation of clinical trial protocol documents and identification of guidelines

#### Prompt structure and execution steps

The prompts used for interacting with ChatGPT are listed in **Table** **2**. The conversation with ChatGPT was structured into four sequential steps: (i) prompt engineering (persona), (ii) summary and analysis, (iii) identifying relevant guidelines, and (iv) eliciting evaluation.

**Table 2:** Prompts used for the evaluation of SAPs and PK–PD methods with ChatGPT, and for the rubric‐based evaluation of the SAPs with ChatGPT and Grok

Prompts for evaluation SAPs & PK–PD methods with ChatGPT		
Prompt #	Prompt	Purpose
	Prompt engineering (PE)	
A1	You are an expert at United States Food and Drug Administration (FDA) with 20 years of regulatory experience reviewing clinical trial protocols	To establish ChatGPT’s role as a regulatory expert for context accuracy
A2	Analyze and summarize the attached clinical trial protocol	To obtain a comprehensive summary and analysis of the provided protocol
	Identifying relevant guidelines	
A3	Obtain pdf files of drug regulatory agencies guidelines for evaluating the statistical analysis plan of clinical trial protocols	To gather standard regulatory documents for statistical analysis plan (SAP) evaluation
A4	Obtain pdf files of drug regulatory agencies guidelines for evaluating the utilization of pharmacokinetics and pharmacodynamics methods in clinical trial protocols	To source regulatory guidelines on PK/PD methodology in clinical trials
	Eliciting evaluations	
A5	Based on the guidelines from the regulatory agencies, evaluate the statistical analysis plan for the following clinical trial protocol	To assess the compliance and quality of the SAP based on regulatory standards
A6	Based on the guidelines from the regulatory agencies, evaluate the utilization of pharmacokinetics and pharmacodynamics methods for the following clinical trial protocol	To evaluate the PK/PD methodology’s adherence to regulatory expectations

Prompts for rubric‐based evaluation of the SAPs with ChatGPT and Grok	
Prompt #	Prompt
B1	You are an expert at the United States Food and Drug Administration (FDA) with 20 years of regulatory experience reviewing clinical trial protocols, and performing the analysis of clinical protocol and SAP
B2	Analyze and summarize the attached clinical trial protocol and statistical analysis plan
B3	You are a senior biostatistician with regulatory experience at a global drug agency. Your task is to evaluate the following response to a clinical trial SAP. Use the grading rubric below to assess the accuracy, completeness, and regulatory alignment of the analysis. Please score each section independently using the rubric, and provide your reasoning. The original SAP is included after the response for reference

The interaction began by establishing ChatGPT’s role as a regulatory expert to ensure context accuracy (Prompt A1, **Table** **2**). Following this, ChatGPT was tasked with generating a comprehensive summary and analysis of the provided clinical trial protocol in Prompt A2, **Table** **2**. A comprehensive summary and analysis of the protocol were generated to provide clinical context, so that SAP and PK–PD sections were not considered in isolation.

After completing the summarization and analysis, ChatGPT was prompted to retrieve standard regulatory documents relevant to evaluating statistical analysis plans (SAPs) and pharmacokinetics/pharmacodynamics (PK–PD) methodologies (Prompts A3 and A4, **Table** **2**).

ChatGPT was provided with the guidelines and prompted to assess the compliance and quality of the SAP and PK–PD analysis plan in the clinical trial protocol based on the regulatory standards (Prompts A5 and A6, **Table** **2**). During this evaluation, all protocol guidelines previously provided by ChatGPT were downloaded and attached to ensure accuracy and consistency.

### Evaluation of SAPs and PK–PD analysis plans

#### Evaluation of SAPs

The prompt used to obtain a structured evaluation of each protocol’s SAP was: From the provided Statistical Analysis Plan, identify the primary efficacy outcome(s), describe the statistical procedure used to evaluate the primary efficacy outcome(s). Include the analysis methods and planned interpretation. Then, assess the appropriateness of the approach based on the FDA E9 Statistical Principles for Clinical Trials.

In Step 1, the prompt obtains the primary efficacy and/or safety outcomes(s), planned statistical procedures, and summarizes the methods of analysis and interpretation. Step 2 was an assessment based on the *FDA E9 Guidance for Industry: Statistical Principles for Clinical Trials* guidance, which includes considerations, such as pre‐specification of end points, handling of multiplicity, choice of analysis population, and robustness of planned inferential techniques.

#### Grading rubric evaluation of ChatGPT SAP outputs by ChatGPT and Grok

Systematic summative evaluations of the SAP outputs from ChatGPT were conducted with ChatGPT and Grok. Grok was included as an additional LLM to mitigate any potential bias from the ChatGPT self‐assessment.^14^ The grading rubric in **Table** **S4** was employed for both ChatGPT and Grok. The criteria were based on the accuracy of primary outcome identification, correctness of statistical methodology, FDA E9 compliance assessment, and clinical interpretability.

The inputs to ChatGPT and Grok were the ChatGPT response, SAP, and *FDA E9 Guidance for Industry: Statistical Principles for Clinical Trials* guidance documents. ChatGPT and Grok were prompt‐engineered with the persona of an FDA expert (Prompt B1, **Table** **2**), and the grading rubric of **Table** **S1** was input with Prompt B3.

#### Evaluation of the PK–PD analysis plans

The ChatGPT PK–PD outputs were manually compared (author E.S.) to the information in the clinical trial protocol. The correctness of the PK and PD objectives and measures, as well as the PK analysis methods, was assessed.

## RESULTS

### Overview of clinical trials selected for assessment

**Tables** **S1** **–** **S3** summarize the trial title, NCT number, condition/disease, intervention/treatment, primary outcome, and locations, obtained from clinicaltrials.gov, for the selected small‐molecule, biological, and global clinical trial protocols.

The median protocol and SAP document set contained 178 pages (minimum: 53 pages, maximum: 620 pages). Trials S3 and S4 had primary safety outcomes only; Trial G5 had primary efficacy and safety outcomes. All other Trials (S1, S2, S5, B1–B5, G1–G4) had primary efficacy outcomes only.

### Evaluation of study design attributes and protocol summary

**Table** **3** is an enumerative and graphical summary of the ChatGPT responses concordant or discrepant relative to the content in the original clinical trial protocol and ClinicalTrials.gov.

**Table 3:** Evaluation of study design attributes of the clinical trial protocols. The first entry in each cell indicates the outcome of the direct comparison to the trial protocol, and the second entry indicates the clinicaltrials.gov comparison

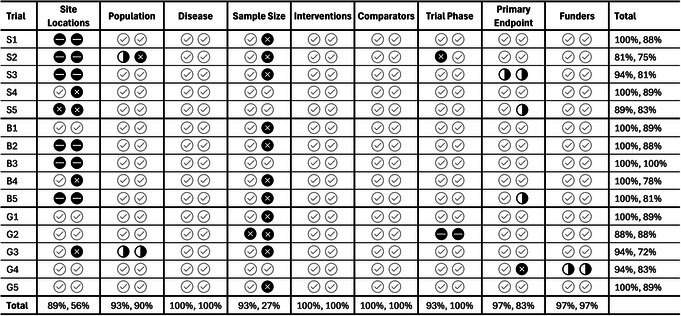

S1–S5 refers to the small‐molecule trials, B1–B5 to the biological trials, and G1–G5 to the global trials. LLM output was graded using the following criteria: Accurate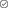: LLM results match both the protocol and clinicaltrials.gov sources; Incorrect 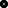: LLM results are inaccurate; Partially Incorrect: 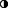 LLM response is missing information; Missing: 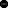 Protocol is missing Information.

ChatGPT identified the Disease, Intervention, and Comparator groups accurately for all 15 trials in the testbed.

The “Protocol is missing information” classification occurred for the Site Locations column of six trials, S1–S3, B2, B3, and B5, because the underlying clinical trial protocol document did not specify site and location details. The exact reasons for the absence of this information in the protocol are not known, but it is likely because individual sites were recruited after the study received approval and/or funding. ChatGPT correctly identified the sites and locations for eight of the nine remaining trials. However, ChatGPT incorrectly identified the site locations for Trial S5 as a single site in Japan when the protocol indicated a single site in the United States. We attribute this error to the nature of the study population: healthy adult subjects of Japanese descent vs. matched, healthy, adult non‐Hispanic Caucasian subjects.

ChatGPT correctly identified the sample size for 14 of 15 trials based on the clinical trial protocols. Discrepancies were noted relative to the ClinicalTrials.gov entries; all discrepancies arose because the ClinicalTrials.gov entries are based on actual enrollments after trial completion. For example, the enrollments specified in the protocols for Trials S1 and S2 were *n* = 150 and approximately *n* = 50 participants, but the actual enrollments in ClinicalTrials.gov were *n* = 159 and *n* = 38, respectively. ChatGPT incorrectly identified the sample size for Trial G2, and its response, “*Not explicitly found in the extracted text; let me know if you want me to search further for it*,” did not match the *n* = 34,000 value of the protocol or the *n* = 58,747 subjects in ClinicalTrials.gov.

ChatGPT correctly identified the trial phase for 14 of the 15 trials. For S2, which was clearly a phase II trial, the ChatGPT response incorrectly indicated, “The document does not explicitly mention the phase. However, based on similar investigational trials, it is likely a Phase 2 or 3 study. Please confirm from the trial registry if needed.”

The primary outcome measure and the funder were correctly identified for 14 of the 15 trials. For G4, the ChatGPT response, “Popular Pharmaceutical Ltd. provided drugs and placebo but had no role in study design, execution, or data access” was marked as partially responsive since the funder was not provided in the protocol.

The overall correctness of the ChatGPT outputs based on the underlying clinical trial protocol documents was 95%, and 80% based on ClinicalTrial.gov.

### Identification of regulatory guidelines for evaluating SAPs and PK–PD analysis plans

#### Regulatory guidelines for SAPs

ChatGPT was prompted to identify the applicable regulatory documents for evaluating the SAP for each of the 15 clinical trial protocols in the test bed.

ChatGPT identified 25 distinct guidelines (**Table** **4**). The most frequently cited guideline was the FDA E9 Statistical Principles for Clinical Trials, identified for 12 out of 15 trials (S1, S3–S5, B1–B3, B5, and G1–G4), followed by the EMA version of the ICH E9 Statistical Principles for Clinical Trials (11/15 trials: S1, S3–S5, B2, B3, B5, G1, G2, G4, and G5), and the PMDA‐Basic Principles on SAP for Clinical Trials, which provides Japan‐specific guidance (5/15 trials: S4, S5, B2, B3, and G1).

**Table 4:** Summary of guidance documents identified for the statistical analysis plans

Order	Guidance identified	Trial	Total/15
1	FDA E9 Statistical Principles for Clinical Trials	S1, S3–S5, B1–B3, B5, G1–G4	12
2	ICH E9 Statistical Principles for Clinical Trials	S2, B4, G1, G3, G4	5
3	EMA ICH E9 ‐Statistical Principles for Clinical Trials	S1, S3–S5, B2, B3, B5, G1, G2, G4, G5	11
5	PMDA‐Basic Principles on SAP for Clinical Trials	S4, S5, B2, B3, G1	5
6	Guidelines for the Content of Statistical Analysis Plans in Clinical Trials (Journal of American Medical Association)	G2, G3	2
7	NIAID: SAP Policy	B4, G2, G3	3
8	NMPA‐Guideline on Data Management Plan and SAP of Drug Clinical Trials	S4, B1	2
9	ICH E3 Structure and Content of Clinical Study Reports	G5	1
12	FDA E9 (R1) Statistical Principles for Clinical Trials: Addendum: Estimands and Sensitivity Analysis in Clinical Trials	G5	1
13	ICH E9 (R1) Addendum on Estimands and Sensitivity Analysis in Clinical Trials to the Guideline on Statistical Principles for Clinical Trials	S1, S2	2
14	EMA ICH E9 (R1) Addendum on Estimands and Sensitivity Analysis in Clinical Trials to Guideline on Statistical Principles for Clinical Trials	S3	1
15	EMA Guideline on Adjustment for Baseline Covariates in Clinical Trials	B4	1
16	FDA ‐ Multiple Endpoints in Clinical Trials	S2	1
17	EMA Guideline on Multiplicity Issues in Clinical Trials	S2, S3	2
18	ICH E10 Choice of Control Group and Related issues in Clinical Trials	G1	1
19	FDA Adaptive Designs for Clinical Trials of Drugs and Biologics	S3	1
20	EMA Guideline on Data Monitoring Committees	B1	1
21	EMA‐ICH E3 Structure and Content of Clinical of Clinical Study Reports	B5	1
22	WHO ‐ Good Clinical Practices^a^	G1	1
23	Health Canada ‐ Guidance for Clinical Trial Sponsors: Clinical Trial Applications	G1	1
24	EMA Committee for Medical Products for Human Use	G5	1
25	PMDA ‐ Basic Principles on Global Clinical Trials	B1, G2–G4	4

S1–S5 are small‐molecule trials, B1–B5 are biological trials, and G1–G5 are global trials. EMA, European Medicines Agency; FDA, United States Food & Drug Administration; ICH, International Council for Harmonisation of Technical Requirements for Pharmaceuticals for Human Use; NMPA, National Medical Products Administration (China); PMDA, Pharmaceuticals and Medical Devices Agency (Japan); WHO, World Health Organization.

^a^ The ChatGPT link did not direct to the WHO Good Clinical Practices handbook.

ChatGPT also cited the ICH E9 (R1) Addendum on Estimands and Sensitivity Analysis (2/15 trials: S1 and S2), highlighting its awareness of statistical frameworks that address estimands and sensitivity analyses, as emphasized in ICH E9(R1).

ChatGPT identification of multiplicity‐related guidelines, such as the EMA Guideline on Multiplicity Issues in Clinical Trials (2/15 trials: S2 and S3) and FDA Guidance on Multiple Endpoints (1/15 trials: S2) was notable. We confirmed that Trials S2 required hierarchical testing to control Type I error (**Table** **4**). However, the omission of multiplicity‐focused guidelines for other trials (e.g., S5 had three dose levels, B1 and B4: three arms, B2, four arms; and G1: nine arms) suggests inconsistency. The selection of EMA Guideline on Adjustment for Baseline Covariates (1/15 trials) for Trial B4 indicates that covariate adjustment was considered.

Overall, ChatGPT identified core SAP guidelines derived from the ICH E9 (e.g., either ICH E9 itself, the FDA version of ICH E9, or the EMA version of ICH E9) with all the trials receiving at least one relevant citation (**Table** **4**). However, there was a lack of specificity in some global health trials where ChatGPT cited broader GCP guidelines (e.g., ICH E3 Structure and Content of Clinical Study Reports for G5, WHO GCP for G1) rather than SAP‐specific documents. For G1, ChatGPT provided a list of global regulatory sources (FDA, EMA, ICH, Health Canada, WHO, and PMDA). ChatGPT also identified some guidelines that were not directly relevant, for example, EMA Guideline on Data Monitoring Committees (1/15 trials: B1) and the WHO Good Clinical Practices (1/15 trials: G1, with a broken link). This suggests occasional overreach or errors in guideline selection.

#### Regulatory guidelines for PK–PD analysis plans

**Table** **S5** summarizes ChatGPT’s identification of guidelines for PK–PD plans for the 15 clinical trial protocols. Across the testbed, ChatGPT identified 29 distinct guidelines in three broad categories of PK–PD, good clinical practices, and clinical pharmacology.

There was an emphasis on population PK–PD, as evidenced by the frequent citation of the FDA Guidance for Industry: Population Pharmacokinetics (12/15 trials: S1, S3–S5, B2–B5, and G2–G5). This guideline focuses on study design and data analysis for population PK studies and is relevant for trials, such as G2–G5 involving diverse populations requiring individualized dosing insights. The EMA Guideline on the Investigation of Drug Interactions was cited in 7/15 trials (S1, S5, B5, and G1–G4), reflecting ChatGPT’s inclusion of drug interaction studies in PK–PD assessments.

The EMA Guideline on the Use of PK–PD in the Development of Antimicrobial Medicinal Products was cited in 6/15 trials (S2, S4, B1, B3, G2, and G5), aligning well with trials involving antimicrobial interventions, such as G2. ChatGPT also identified ICH guidelines, such as ICH E4 Dose–Response Information (2/15 trials: G1 and G5) and EMA ICH E8(R1) General Considerations for Clinical Studies (3/15 trials: S2, B5, and G3), indicating an awareness of broader clinical trial design principles that intersect with PK–PD methodologies.

However, inconsistencies emerged, such as the citation of less relevant guidelines like ICH E6(R2) Good Clinical Practice (1/15 trials: S2) and the inclusion of non‐functional links, such as the EMA Guideline on Reporting PK Studies for S3 and WHO Good Clinical Practice Guidelines for S5 and G1. The global health trials (G1–G5) showed a higher incidence of broad citations (e.g., G1 listing FDA, EMA, ICH, Health Canada, WHO).

### Evaluation of the SAPs

#### Statistical procedures for primary efficacy and safety outcomes

**Table** **5** shows synopses of ChatGPT evaluations of the statistical procedures for six trials (S1, S2, B1, B2, G1, and G2); the synopses for all 15 trials are in **Table** **S6**. The ChatGPT outputs were clear and structured.

**Table 5:** Synopsis of the ChatGPT assessment of statistical analysis plans

Trial	Primary outcome	Statistical method	Planned interpretation	E9 compliance
S1	End point: PFS per RECIST 1.1, abemaciclib vs. docetaxel in Stage IV squamous NSCLC (SAP Section 4.1)	Primary: Bayesian model, historical data, 120 PFS events. Supplementary: Log‐rank, Kaplan–Meier, Cox model (SAP Section 6.7.2)	HR < 1 (target 0.64, 90.5% power, *α* = 0.05); sensitivity analyses (SAP Section 6.7.2)	Prespecified, ITT, robust; lacks sensitivity analysis detail, open‐label bias risk (SAP Section 6.7.2)
S2	End point: Clinical response at Week 12 via modified Mayo score (stool frequency, rectal bleeding, endoscopy, PGA)	Primary: Clopper–Pearson for 95% CI. Supportive: Logistic regression for odds ratio. Missing Data: Nonresponder imputation; sensitivity analyses planned	Response rate ~60% vs. 33% placebo, targeting superiority	Prespecified endpoint, robust methods, justified imputation; lacks covariate, sensitivity analysis details (ICH E9 Sections II.B.2, V.C, V.E, V.G)
B1	End points: PFS and OS in advanced melanoma, comparing pembrolizumab vs. ipilimumab (SAP Section 3.5.3.1)	Methods: Stratified log‐rank test, Kaplan–Meier curves, Cox model for HR (stratified by PD‐L1 expression); hierarchical testing for multiplicity; interim analyses with Lan‐DeMets O’Brien‐Fleming boundaries (SAP Section 3.5.3.1)	Significant PFS/OS improvement indicates pembrolizumab benefit; HR and CIs assess effect size; exploratory subgroup analyses by PD‐L1 status (SAP Section 3.5.3.1)	Prespecified end points, ITT analysis, multiplicity control, robust methods; needs detailed sensitivity analyses and cautious subgroup interpretation (ICH E9 Sections 2.3, 5.1, 5.2, 5.5, 5.6)
B2	End point: Treated annualized bleed rate (ABR) in Hemophilia A patients without FVIII inhibitors, for emicizumab (1.5 mg/kg weekly, 3 mg/kg biweekly) vs. no prophylaxis (SAP Section 2.3.1)	Method: Negative binomial regression with treatment group covariate, bleed count as dependent variable, log of observation time offset, addressing overdispersion (SAP Section 2.3.1)	Rate ratios with 95% CIs and *P* values; significant bleed rate reduction indicates emicizumab efficacy (SAP Section 2.3.1)	Prespecified end point, ITT analysis, suitable negative binomial model, stratified randomization minimizes bias, clinically relevant, robust with sensitivity analyses (ICH E9 Sections 2.3, 3.5, 5.1, 5.2, 5.3, 5.5, 5.7)
G1	End point: Early Bactericidal Activity (EBA), slope of log_10_ CFU/mL sputum change from baseline to Day 14, assessed in Stages 1 and 2 (SAP Sections 6.1.1, 8.1.1)	Methods: Two‐time point EBA slope; linear mixed‐effects model (fixed: time, treatment, interaction; random: subject intercepts); Fieller’s and Taylor’s (Delta) methods for EBA ratio CIs (SAP Sections 6.1.1, 8.1.1)	EBA slope indicates bactericidal activity; differences vs. RHEZ control show OPC‐167832 efficacy; exposure‐response modeling supports effect (SAP Sections 6.1.1, 8.1.1)	Prespecified EBA, robust mixed model, valid CI methods, appropriate longitudinal handling, clinically relevant, no multiplicity issues (ICH E9 Section 5)
G2	End points: Maternal death/sepsis (42 days postpartum) and neonatal death/sepsis (28 days postpartum) (SAP not specified)	Populations: ITT (primary), High‐Risk Cohort, As‐Treated (sensitivity). Method: Generalized Linear Model (log link) for relative risk (RR) with 95% CIs, adjusted for site, robust standard errors for clustering; no multiplicity adjustment (SAP not specified)	Significant RR < 1 (95% CI excluding 1) supports azithromycin efficacy; independent evaluation of each end point (SAP not specified)	Prespecified end points, ITT analysis, randomization, robust GLM; partial concern for multiplicity without formal adjustment, needs clarification (ICH E9 Sections not specified)

In B1, it identified progression‐free survival (PFS) and overall survival (OS) as primary efficacy end points, outlined statistical methods, including Kaplan–Meier and Cox models, and provided interpretation aligned with the SAP’s risk–benefit framework. In S5, it correctly identified PK parameters as the primary “efficacy” outcome for a phase I trial and accurately detailed statistical methods, including ANOVA and power model analysis. These examples highlight ChatGPT’s ability to extract and summarize technical details from well‐defined SAPs, making it a valuable tool for trial analysis.

Some limitations with specificity and contextual accuracy were found. For example, in B3, the ChatGPT output stated that the objective response rate (ORR) was assessed by an Independent Review Committee (IRC), whereas the updated SAP (Version 2.0) specified an investigator‐assessed ORR; the Kaplan–Meier curves for OS. In G5, it misstated a two‐sided test for vaccine efficacy instead of the one‐sided test specified; it omitted the Kruskal–Wallis test for safety. In G4, it failed to identify end‐point components, such as early/late clinical improvement and WHO criteria, and missed the subgroup analyses.

#### Grading rubric evaluation of ChatGPT SAP outputs by ChatGPT and Grok

**Figure 1:**
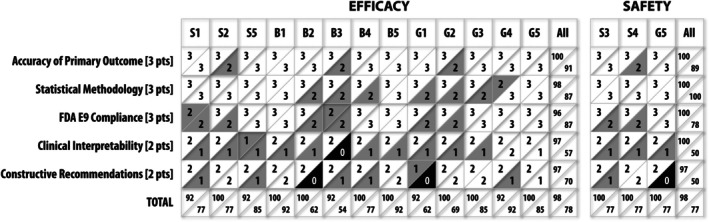
Results ChatGPT and Grok assessment of the ChatGPT responses for efficacy and safety of the clinical trial protocols using the evaluation rubric. Trials S3 and S4 had primary safety outcomes only, Trial G5 had primary efficacy and safety outcomes; all other Trials (S1, S2, S5, B1–B5, G1–G4) had primary efficacy outcomes only. The ChatGPT evaluations are shown in the upper triangle region, and the Grok results are in the lower triangle region of each cell. The Accuracy of Primary Outcome, Statistical Methodology, and FDA E9 Compliance each had a maximum of 3 points, and the Clinical Interpretability and Constructive Recommendation criteria had a maximum of 2 points each. The average percentages for each row and column are also shown. The white color indicates a maximum score, the gray color indicates 1 point less than maximum, and black indicates 2 points less than maximum.

**Figure** **1** shows the results from the self‐evaluation of SAP responses by ChatGPT and Grok. The outputs were evaluated on a rubric for the Accuracy of Primary Outcome Identification, Statistical Methodology Correctness, FDA E9 Compliance Assessment, Clinical Interpretability, and Constructive Recommendations. The sub‐scores assigned by Grok were generally lower than those of ChatGPT. The Accuracy of Primary Outcome Identification, Statistical Methodology Correctness, and FDA E9 Compliance Assessment scores were qualitatively similar for ChatGPT and Grok. However, the deviations in the Clinical Interpretability (95% for ChatGPT vs. 53.3% for Grok) and Constructive Recommendations (93.3% for ChatGPT vs. 68.3% for Grok) scores were more pronounced. The discrepant scores indicate the challenges of scoring the quality of subjective assessments in clinical trial protocols.

### Evaluation of PK–PD analysis plans

PK–PD analyses were presented in nine clinical trial protocols: S1, S3, S5, B1–B4, G1, and G2. **Table** **6** shows the synopsis of the ChatGPT results of PK–PD analyses in the trials with PK–PD components. The PK objectives and measures were correctly identified for eight of the nine trials. However, for B2, ChatGPT incorrectly included peak concentrations when the protocol did not have post‐dose sampling.

**Table 6:** Synopsis of the ChatGPT assessment of PK–PD analysis plans

Trial	PK objectives & measures	PD objectives & measures	PK analysis method
S1	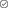 “Plasma concentrations of Abemaciclib and its active metabolites…”	“ 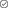 … as well as linking neutrophil and lymphocyte counts.”	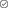 NONMEM for PK/PD analysis with covariate effects (e.g., age, sex, plasma protein levels)
S3	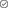 “Cmax, Tmax, AUClast, AUCinf, CL/F, and Vz/F for plasma PK analysis…” “Urinary PK parameter (e.g., Ae%, CLr) are…”	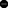	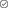 NCA analysis
S5	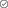 “Key PK parameters include Cmax, AUC, Tmax, and terminal half‐life…”	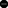	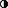 “ANOVA for comparing PK parameters…” Protocol has ANOVA and NCA
B1	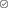 “Trough (pre‐dose) and peak (post‐dose) samples are collected at specific cycles…”	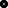 PD‐L1 (Programmed Death‐Ligand 1), a biomarker used for stratification was conflated with pharmacodynamics (PD)	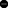
B2	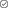 “The Phase III study (Protocol BH30071) evaluates emicizumab PK through trough levels and time‐dependent concentration profiles”	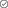 “The primary endpoint (treated bleed rate) will be analyzed in relation to emicizumab exposure”	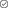 PopPK/PD models
B3	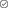 “The protocol schedules regular PK sampling, …”	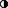 “**Exposure‐Response Analysis:** Investigating the relationship between nivolumab concentration and clinical outcome…” Summary statistics for biomarkers of immunoregulatory activity not included	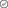 PopPK/PD models. “**Covariate Analysis:** The plan to analyze the effects of covariates (e.g., weight, age, biomarkers)…”
B4	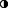 “The protocol specifies multiple PK sampling points during the trial (e.g., pre‐dose, peak concentration, and steady state).…” No post‐dose sampling for peak concentration	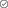 “PD endpoints, such as changes in inflammatory markers (CRP, ESR) are integrated…”	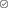 PopPK/PD models
G1	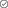 “PK parameters like Cmax, AUC, Tmax and terminal half‐life, …”	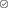 “…PD measures like QT interval relationships and bacterial load, …”	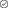 PopPK/PD models
G3	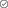 “Plasma concentration‐time profiles (PK).”	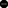	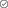 PopPK/PD models

Excerpts from the ChatGPT response are in quotes; other clarifications are not in quotes. S1–S5 refers to the small‐molecule trials, B1–B5 to the biological trials, and G1–G5 to the global trials. LLM output was graded using the following criteria: Accurate 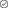 : LLM results matched the protocol; Incorrect 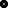 : LLM results are inaccurate; Partially Incorrect 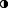 : The LLM response is missing information; Missing 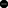 : Protocol is missing Information.

Six of the nine protocols with PK had PD objectives and measures. ChatGPT correctly identified the PD objectives and measures for four of the six protocols. B1 did not have a PD end point, but ChatGPT erroneously conflated PD‐L1 (Programmed Death‐Ligand 1), a biomarker used for stratification in the trial, as a PD measure. For trial B3, ChatGPT did not include immunoregulatory activity biomarkers that were considered for PD.

The PK analysis methods were correctly identified for seven of the eight trials. S3 and S5 employed non‐compartmental analysis, whereas the remaining six trials (S1, B2–B4, G1, and G2) used population PK analysis. The ChatGPT response for S5 was marked incomplete because the protocol used ANOVA and non‐compartmental analysis.

The ChatGPT assessment of Trial S5, whose primary outcomes were the PK end points $C_{\max}$ and AUC of maribavir following single oral doses of 200, 400, and 800 mg, was viewed as insightful. It correctly inferred a standard dose proportionality interpretation (Slope β≈1 in the power law model $\ln\;\left(\mathrm{PK}\;\text{Parameter}\right)=\alpha +\beta \times \ln\;\left(\text{Dose}\right)+\text{Random error}$),^15^ even though the SAP only implied the equation. ChatGPT also interpreted the β parameter confidence intervals (CI) by assessing whether the 90% CI was within the $\left(1+\ln\left(0.8\right)/\ln r\right)$ and $\left(1+\ln\left(1.25\right)/\ln r\right)$ interval, adjusted for the dose ratio r. ^16^

## DISCUSSION

In this research, we evaluated ChatGPT’s performance for reviewing clinical trial protocols. ChatGPT’s performance was assessed using a testbed of 15 clinical trials across diverse therapeutic areas and pharmacological classes. The assessment tasks included extracting study design attributes, identifying relevant regulatory SAP and PK–PD guidelines, and providing a detailed evaluation of the statistical testing procedures in the SAP.

We deliberately opted not to use LLM to evaluate every aspect of a clinical trial protocol, reasoning that the broad scope would present unexpected challenges in evaluating LLM performance. Instead, a systematic focus on a few critical protocol dimensions, such as study design attributes, SAP, and PK–PD analysis plans, could enable informed expansion to larger portions of protocols later. As noted previously, study design attributes provide useful context for clinical trial protocol reviewers. SAPs were selected because they are ubiquitous in protocols and can be objectively evaluated. Although PK–PD undergirds rational dose determination, it is generally not included or is inadequately considered in most clinical trial protocols.

Burford *et al*. analyzed reviewer recommendations for 52 clinical trial protocols submitted to the Bill and Melinda Gates Foundation (BMGF), a major funder of clinical trials worldwide. The BMGF reviewers made a median of 28 recommendations per protocol (Range: 13–52), with 47% of recommendations categorized as high priority. Recommendations related to statistics and data analysis, trial procedures, and intervention/dose occurred with the highest frequencies.^17^ These considerations motivated our decision to focus on the SAP and PK–PD aspects with ChatGPT.

As a precursor to SAP and PK–PD analysis, we obtained a full protocol summary and analysis instead of providing only the SAP and PK–PD sections as input. This step‐by‐step process enables direct input of available clinical protocol documents and, more importantly, it provides adequate clinical context to inform SAP and PK–PD analysis. We evaluated ChatGPT results with Grok. By design, Grok was not informed that the input was obtained from ChatGPT’s response. We considered DeepSeek but could not upload the protocol documents, which were > 300 pages in some cases, as input. The tone of the Grok responses differed from those of ChatGPT. Grok provided elaborate responses annotated with source information, whereas ChatGPT was succinct and informative. In the research evaluation setting, both types of output responses are complementary.

We also evaluated the context in which ChatGPT provided insightful findings and made errors to gain a better understanding of its strengths and limitations in evaluating clinical trial protocols.

In Trial B3, ChatGPT incorrectly reported that an IRC evaluated the two co‐primary end points (overall response rate, or ORR, and overall survival or OS) based on information from the outdated SAP version 2.0. The updated SAP (v03) included a note at the end of the protocol indicating that the primary end point was changed to OS as the sole end point and that ORR was determined by the investigator, not by the IRC. Errors of this type may also be challenging for some human reviewers.

For Trial G5, the ChatGPT response erroneously reported a two‐sided vaccine efficiency test, missing the design’s adaptive one‐sided test for interim monitoring. However, Trial G5, a study evaluating a canarypox‐protein HIV vaccine (ALVAC‐HIV plus bivalent subtype C gp120/MF59), had a hybrid phase IIb/III design, incorporating proof‐of‐concept and confirmatory elements, cohort structures, staged analyses, and adaptive monitoring.^18^ The G5 case underscores the importance of enhanced human review of LLM reviews of phase IIb/III designs.

The limitations of our study design and the LLM approach should be considered. Investigational new drug (IND) application information is confidential between the FDA and the sponsor. Therefore, clinicaltrials.gov collects but does not publicly release IND information. Having access to protocols that were not accepted initially and required revisions would have strengthened our assessments of non‐compliance. While SAP guidelines derived from the ICH E9, that is, either ICH E9 itself, or the FDA or EMA versions of ICH E9, were identified for all 15 trials, FDA E9, which appears applicable to all SAPs, was identified only for 12/15 trials. We did not limit our guidance document prompt to focus on FDA resources, and the variability is attributable to homologous content in SAP documents from different regulatory agencies due to the adoption of ICH E9 SAP guidance. The limitations of the LLM approach include concerns about data security when uploading proprietary or confidential information, performance variations attributable to the LLM model selected, and the difficulties in creating effective prompts.

In conclusion, our results demonstrate the performance, strengths, and weaknesses of ChatGPT in various tasks related to evaluating clinical trial protocols. ChatGPT’s performance on the study design attributes, broader regulatory context, and technical statistical methodology tasks was satisfactory. ChatGPT could be useful as an aid in a human‐in‐the‐loop strategy for reviewing clinical trial protocols in high‐volume settings, such as those of regulatory and funding agencies. Additional well‐controlled validation studies are warranted, given the promising results we have obtained.

## FUNDING

This work was funded by INV‐080729 from the Design, Analyze, Communicate Integrated Development Global Health Division of the Bill and Melinda Gates Foundation. We gratefully acknowledge support for the Ramanathan laboratory from Award MS190096 from the Department of Defense Congressionally Directed Medical Research Programs, USAMRDC, Multiple Sclerosis Research Program. The funders had no role in the study’s design or data analysis.

## CONFLICT OF INTEREST

The authors declared no competing interests for this work.

## AUTHOR CONTRIBUTIONS

M.R. designed the research. All authors wrote the manuscript and analyzed the data.

## USE OF ARTIFICIAL INTELLIGENCE

Artificial intelligence tools, such as ChatGPT and Grok, were utilized to conduct the research. AI features in Grammarly and Microsoft Word were used to enhance readability and language; tools such as Google and EndNote were employed to curate references, add citations, and generate the bibliography, and Illustrator was used to draw figures.

## Supporting information


**Data S1**Supporting Information.

**Data S2**Supporting Information.
