# Supplementary material for: Large Language Models for Clinical Trial Protocol Assessments
Source: Clin Pharmacol Ther. 2025 Oct 21;119(2):393–402. doi: 10.1002/cpt.70096 (PMC12816432; doi:10.1002/cpt.70096)
Supplement: Supplementary file 1 — Data S1Supporting Information. [file CPT-119-393-s001.pdf]

SUPPLEMENTARY FILE

Evaluation of Large Language Models for Clinical Trial Protocol Analysis

Euibeom Shin, Amruta Gajanan Bhat, and Murali Ramanathan

Department of Pharmaceutical Sciences, University at Buffalo, The State University of New York, Buffalo, NY, USA.

CORRESPONDING AUTHOR: Murali Ramanathan
355 Pharmacy, Department of Pharmaceutical Sciences
State University of New York, Buffalo, Buffalo, NY 14214-8033.
(716)-645-4846 and FAX 716-829-6569. E-mail Murali@Buffalo.Edu

Running Head: Large Language Models for Clinical Trial Protocol Analysis

Table S1. Information on the small-molecule clinical trials selected for evaluation.

<table>
<tr><th colspan="3">SMALL-MOLECULE CLINICAL TRIALS</th></tr>
<tr>
<td>Small-Molecule Trial 1. A Randomized Phase 2 Study of Abemaciclib (LY2835219) versus Docetaxel in Patients with Stage IV Squamous Non-Small Cell Lung Cancer Previously Treated with Platinum Based Chemotherapy
NCT Number: NCT02450539
Condition/Disease: Stage IV Squamous non-small cell lung cancer
Intervention/Treatment: Abemaciclib vs. Docetaxel
Primary Outcome Measure: Progression-free survival
Location: Multicenter, USA
Protocol: https://cdn.clinicaltrials.gov/large-docs/39/NCT02450539/Prot_002.pdf
SAP: https://cdn.clinicaltrials.gov/large-docs/39/NCT02450539/SAP_001.pdf</td>
<td>Small-Molecule Trial 2. A Phase 2 Randomized, Double-Blind, Placebo-Controlled Study of the Safety, Efficacy, and Biomarker Response of BMS-986165 in Subjects with Moderate to Severe Ulcerative Colitis
NCT Number: NCT04613518
Condition/Disease: Ulcerative colitis
Intervention/Treatment: BMS-986165 vs. Placebo
Primary Outcome Measure: Clinical response at Week 12
Location: Multicenter, USA</td>
<td>Small-Molecule Trial 3. Phase 1 Study of PF-07321332 in Healthy Participants
NCT Number: NCT04756531
Condition/Disease: Healthy volunteers
Intervention/Treatment: PF-07321332
Primary Outcome Measure: Safety and pharmacokinetics
Location: USA</td>
</tr>
<tr>
<td>Small-Molecule Trial 4. Reldesemtiv in Amyotrophic Lateral Sclerosis (ALS): A Phase 3, Open-Label Extension of COURAGE-ALS (CY-5031)
NCT Number: NCT05442775
Condition/Disease: Amyotrophic lateral sclerosis
Intervention/Treatment: Reldesemtiv vs. Placebo
Primary Outcome Measure: Change in ALS Functional Rating Scale
Location: Multicenter, Global
Protocol: https://cdn.clinicaltrials.gov/large-docs/75/NCT05442775/Prot_000.pdf
SAP: https://cdn.clinicaltrials.gov/large-docs/75/NCT05442775/SAP_001.pdf</td>
<td colspan="2">Small-Molecule Trial 5. A Phase 1, Open-label, Randomized, Cross-over Study to Evaluate the Pharmacokinetics, Safety, and Tolerability of a Single Oral Dose of Maribavir Administered in Healthy Japanese Subjects Compared with Matched, Healthy, Non-Hispanic, Caucasian Subjects and to Assess Dose-Proportionality of 3 Doses of Maribavir in the Japanese Subjects
NCT Number: NCT04497883
Condition/Disease: Healthy Volunteers
Intervention/Treatment: Maribavir (single oral doses of 200 mg, 400 mg, and 800 mg)
Primary Outcome Measure: Pharmacokinetic parameters including Cmax (maximum observed concentration) and AUC (area under the concentration-time curve)
Location: United States
Protocol: https://cdn.clinicaltrials.gov/large-docs/83/NCT04497883/Prot_000.pdf
SAP: https://cdn.clinicaltrials.gov/large-docs/83/NCT04497883/SAP_001.pdf</td>
</tr>
</table>

Table S2. Information on the large-molecule clinical trials selected for evaluation.

<table>
<tr><th colspan="3">LARGE-MOLECULE CLINICAL TRIALS</th></tr>
<tr>
<td>Large-Molecule Trial 1. A Multicenter, Randomized, Controlled, Three-Arm, Phase III Study to Evaluate the Safety and Efficacy of Two Dosing Schedules of MK-3475 Compared to Ipilimumab in Patients with Advanced Melanoma**
Product: MK-3475
Protocol/Amendment: 006-04
NCT Number: NCT01866319
Condition/Disease: Advanced melanoma
Intervention/Treatment: Pembrolizumab
Primary Outcome Measure: Overall survival
Location: Multicenter, USA
https://pubmed.ncbi.nlm.nih.gov/25891173/</td>
<td>Large-Molecule Trial 2. A Randomized, Multicenter, Open-Label, Phase III Clinical Trial to Evaluate the Efficacy, Safety, And Pharmacokinetics of Prophylactic Ro5534262 Versus No Prophylaxis in Hemophilia A Patients with Inhibitors
IND Number: 122,954
Protocol Number: BH29884
Condition/Disease: Hemophilia A
Intervention/Treatment: Emicizumab (RO5534262)
Primary Outcome Measure: Bleeding events rate
Location: Multicenter, USA
Protocol: https://cdn.clinicaltrials.gov/large-docs/55/NCT03315455/Prot_000.pdf
SAP: https://cdn.clinicaltrials.gov/large-docs/37/NCT02847637/SAP_001.pdf</td>
<td>Large-Molecule Trial 3. Phase 3 Nivolumab versus Docetaxel in Advanced Squamous-Cell Non-Small-Cell Lung Cancer
NCT Number: NCT01642004
Clinical Protocol: CA209017
Condition/Disease: Non-Small Cell Lung Cancer
Intervention/Treatment: Nivolumab
Primary Outcome Measure: Progression-free survival
Location: Multicenter, USA
https://pubmed.ncbi.nlm.nih.gov/26028407/</td>
</tr>
<tr>
<td>Large-Molecule Trial 4. Phase 3, A randomized, double-blind, placebo-controlled, multicenter study of secukinumab to demonstrate the efficacy at 16 weeks and to assess the long-term safety, tolerability and efficacy up to 2 years in patients with active Ankylosing Spondylitis
NCT Number: NCT01358175
Condition/Disease: Ankylosing spondylitis
Intervention/Treatment: Secukinumab
Primary Outcome Measure: ASAS20 Response at Week 16
Location: Multicenter, USA
Protocol & SAP: https://www.nejm.org/doi/suppl/10.1056/NEJMoa1505066/suppl_file/nejmoa1505066_protocol.pdf</td>
<td colspan="2">Large-Molecule Trial 5 A Phase 3 Confirmatory Study Investigating the Efficacy and Safety off Dupilumab Monotherapy Administered to Adult Patients with Moderate-to-Severe Atopic Dermatitis
NCT Number: NCT02277743
Condition/Disease: Atopic dermatitis
Intervention/Treatment: Dupilumab
Primary Outcome Measure: EASI-75 Score at Week 16
Location: Multicenter, USA
Protocol & SAP: https://www.nejm.org/doi/suppl/10.1056/NEJMoa1610020/suppl_file/nejmoa1610020_protocol.pdf</td>
</tr>
</table>

Table S3. Information on the global health clinical trials selected for evaluation.

<table>
<tr><th colspan="3">GLOBAL CLINICAL TRIALS</th></tr>
<tr>
<td>Global Trial 1. A Phase 1/2 Trial of Multiple Oral Doses of OPC-167832 for Uncomplicated Pulmonary Tuberculosis
NCT Number: NCT03678688
Condition/Disease: Pulmonary tuberculosis
Intervention/Treatment: OPC-167832 (Quabodepistat)
Primary Outcome Measure: Safety and tolerability of multiple oral doses
Location: South Africa</td>
<td>Global Trial 2. Safety, Tolerability and PK of Multiple-ascending Doses of Emodepside
NCT Number: NCT02661178
Condition/Disease: Helminth infections
Intervention/Treatment: Emodepside
Primary Outcome Measure: Safety and tolerability of multiple ascending doses
Location: Germany</td>
<td>Global Trial 3. Global Trial 3. Efficacy and Safety of KAF156 in Combination With LUM-SDF in Adults and Children With Uncomplicated Plasmodium Falciparum Malaria
NCT Number: NCT03167242
Condition/Disease: Acute uncomplicated plasmodium falciparum malaria
Intervention/Treatment: 1. KAF156 2. Coartem, 3. Lumefantrine Solid dispersion formulation
Primary Outcome Measure: Number of participants with polymerase chain reaction (PCR)-corrected Adequate Clinical and Parasitological Response (ACPR) at Day 29.
PK Run-in: Area Under the Blood Concentration-time Curve Over the Last 24 Hours After Treatment Dose (AUC0-24h) of KAF156
Location: 11 locations, Burkina Faso, Gabon, India, Kenya, Mali, Mozambique, Thailand, Uganda, Vietnam
Clinical Trial Protocol: https://cdn.clinicaltrials.gov/large-docs/42/NCT03167242/Prot_000.pdf
SAP: https://cdn.clinicaltrials.gov/large-docs/42/NCT03167242/SAP_001.pdf</td>
</tr>
<tr>
<td>Global Trial 4. Clinical Trial of Ivermectin Plus Doxycycline for the Treatment of Confirmed Covid-19 Infection
NCT Number: NCT04523831
Condition/Disease: Mild to moderate COVID-19 infection
Intervention/Treatment: Ivermectin 12 mg (single dose) + Doxycycline 100 mg twice daily for 5 days + Standard care
Primary Outcome Measure: Number of days required for clinical improvement
Location: Bangladesh</td>
<td colspan="2">Global Trial 5 A Pivotal Phase 2b/3 ALVAC/Bivalent gp120/MF59 HIV Vaccine Prevention Safety and Efficacy Study in South Africa (HVTN702)
NCT Number: NCT02968849
Condition/Disease: HIV Infection
Intervention/Treatment: ALVAC-HIV (vCP2438) and bivalent subtype C gp120/MF59
Primary Outcome Measure: HIV Incidence
Location: South Africa</td>
</tr>
</table>

Table S4. Grading rubric used for evaluating SAP assessments with ChatGPT and Grok.

1. Accuracy of Primary Outcome Identification (0-3 points)
Evaluation Factors:
- Correctly identifies **primary efficacy/safety endpoints** from SAP
- Captures **composite endpoints, timepoints, and analysis populations**
- Avoids confusion between primary/secondary outcomes

Grading Examples:
- **3/3**: "Primary efficacy outcome: Progression-Free Survival (PFS) per RECIST 1.1 at Week 24, analyzed in the ITT population." (Fully matches SAP definition)
- **2/3**: "Primary outcome: PFS (no RECIST version or timepoint specified)." (Minor omission)
- **1/3**: "Primary outcome: Tumor response rate." (Incorrect – was secondary in SAP)
- **0/3**: "No primary outcome identified."

2. Statistical Methodology Correctness (0-3 points)
Evaluation Factors:
- Describes **analysis method** (e.g., Cox model, logistic regression)
- Specifies **key details** (stratification, covariates, missing data handling)
- Flags **errors** (e.g., wrong test for endpoint type)

Grading Examples:
- **3/3**: "Primary analysis: Stratified log-rank test for PFS, with HR from Cox model adjusted for PD-L1 status and region." (Matches SAP exactly)
- **2/3**: "Uses Cox model for PFS but omits stratification factors." (Partial accuracy)
- **1/3**: "ANOVA used for time-to-event data." (Wrong method)
- **0/3**: "No statistical method described."

3. FDA E9 Compliance Assessment (0-3 points)
Evaluation Factors:
- Explicitly references **ICH E9 sections** (e.g., 5.1, 6.4)
- Evaluates **bias control, multiplicity, missing data** per E9
- Distinguishes **confirmatory vs. exploratory** alignment

Grading Examples:
- **3/3**: "SAP prespecifies endpoints per E9 Section 5.1. Missing data handled via multiple imputation (E9 Section 5.3)."
- **2/3**: "Notes prespecification but doesn't cite E9 sections."
- **1/3**: "States 'analysis is E9-compliant' without justification."
- **0/3**: "No E9 evaluation provided."

4. Clinical Interpretability (0-2 points)
Evaluation Factors:
- Explains **clinical relevance** of statistical results
- Avoids **overly technical jargon** without context
- Links endpoints to **patient-centric outcomes**

Grading Examples:
- **2/2**: "A 2-month PFS improvement represents clinically meaningful delay in progression for metastatic NSCLC."
- **1/2**: "PFS HR of 0.64 favors treatment." (No clinical context)
- **0/2**: "Statistical significance achieved ($p<0.05$)." (No interpretation)

5. Constructive Recommendations (0-2 points)
Evaluation Factors:

- Suggests **actionable SAP improvements**
- Proposes **regulatory-aligned enhancements**
- Avoids **vague statements**

Grading Examples:

- **2/2**: “Add sensitivity analysis for non-random missing data per E9(R1) Estimands guidance."
- **1/2**: “Consider more sensitivity analyses." (Too generic)
- **0/2**: “No recommendations provided."

6. Aggregate Scoring Example

ChatGPT Response Excerpt	Accuracy (0-3)	Stats (0-3)	E9 (0-3)	Interpretability (0-2)	Recommendations (0-2)	Total
"Primary: ORR (binary). Analysis: CMH test stratified by region. E9: Compliant but lacks multiplicity control. Clinically, ORR correlates with survival in lymphoma. Suggest adding Hochberg adjustment."	3	3	2	2	2	**12/13**
"Safety: AEs summarized descriptively. No stats testing."	2	1	1	1	0	**5/13**

Table S5. Guidance documents identified for the PK-PD analysis plans.

Order	Guidance Identified	Trial	Total/15
1	FDA-Guidance for Industry POPPK	S1, S3-S5, B2-B5, G2-G5	12
2	EMA-Guideline on Reporting the Results of POPPK	S1, S3, S5	3
3	PMDA Guideline on POPPKPD Analysis	B1, B3, G1, G3	4
4	EMA-Guideline on the Investigation/Drug Interactions	S1, S5, B5, G1-G4	7
5	EMA-Guideline on the Use of PK and PD in the Development of Antimicrobial Medicinal Products	S2, S4, B1, B3, G2, G5	6
6	WHO Guidance on PKPD for Antimicrobials	G1	1
7	FDA E-R Relationships- Study Design, Data Analysis and Regulatory Applications	S5, B1, G1, G5	4
8	ICH E4 D-R Info to Support Drug Registration	G1, G5	2
9	EMA ICH E4 Dose-Response Information to Support Drug Registration	S1	1
10	PMDA: Clinical PK Studies/Pharmaceuticals	G3, G4	2
11	EMA- Clinical pharmacology and pharmacokinetics: Q&A Website	S2	1
12	FDA-PK in Patients with Impaired Renal Function - Study, Design, Data, Analysis, and Impact on Dosing Guidance for Industry	S2	1
13	FDA-PBPK Analyses Format and Content	S4	1
14	FDA Bioavailability and Bioequivalence Studies Submitted in NDAs or INDs	S3	1
15	FDA - E8 R1 General Considerations for Clinical Studies	S4, B4	2
16	ICH E8 General Considerations for Clinical Trials	S2	1
17	ICH E6 Good Clinical Practice Consolidated Guidance	B4	1
18	ICH E6 R2 Addendum Guideline for Good Clinical Practice	S2	1
19	EMA ICH E8 R1 on General Considerations for Clinical Studies	S2, B5, G3	2
20	WHO guidelines for good clinical practice GCP for trials on pharmaceutical products: responsibilities of the investigator Article is not Free)	S5, G1	2
21	Health Canada - Guidance for CT Sponsors: CT Applications	G1	1
22	ICH E17- General Principles for Planning and Design/Multi-Regional Clinical Trials	S1	1
23	EMA ICH E14 QT/QTc	S1, G5	2
24	FDA E14 Clinical Evaluation/QT/QTc Interval Prolongation and Proarrhythmic Potential for Non-Antiarrhythmic Drugs	S5	1
25	EMA ICH M3 R2 on non-clinical safety studies for the conduct/human clinical trials and marketing authorisation for pharmaceuticals	S3, B4, G5	3
26	FDA-Clin Pharm Section/Labeling	S1	1
27	FDA-Clin Pharm Early Drug Development	B5	1
28	EMA ICH E5 R1 Ethnic Factors in the Acceptability/Foreign Clinical Data	S5	1
29	Hallucination -EMA Guideline on Reporting PK Studies: • Highlights best practices for designing, analyzing, and reporting pharmacokinetic studies. • Link to download: EMA PK Guideline." Page doesn't exist	S3	1

Table S6. Synopsis of the ChatGPT assessment of statistical analysis plans.

Trial	Primary Outcome	Statistical Method	Planned Interpretation	E9 Compliance
S1	Endpoint: PFS per RECIST 1.1, abemaciclib vs. docetaxel in Stage IV squamous NSCLC (SAP Section 4.1).	Primary: Bayesian model, historical data, 120 PFS events. Supplementary: Log-rank, Kaplan-Meier, Cox model (SAP Section 6.7.2).	HR < 1 (target 0.64, 90.5% power, α = 0.05); sensitivity analyses (SAP Section 6.7.2).	Prespecified, ITT, robust; lacks sensitivity analysis detail, open-label bias risk (SAP Section 6.7.2).
S2	Endpoint: Clinical response at Week 12 via modified Mayo score (stool frequency, rectal bleeding, endoscopy, PGA).	Primary: Clopper-Pearson for 95% CI. Supportive: Logistic regression for odds ratio. Missing Data: Nonresponder imputation; sensitivity analyses planned.	Response rate ~60% vs. 33% placebo, targeting superiority.	Prespecified endpoint, robust methods, justified imputation; lacks covariate, sensitivity analysis details (ICH E9 Sections II.B.2, V.C, V.E, V.G).
S3	Endpoints: TEAEs (frequency, severity, causality, withdrawals), clinical laboratory abnormalities, vital signs, and ECG changes (focus on QTcF) across all study parts (SAP Sections 3.5.1–3.5.4; Protocol Sections 1.1, 3).	Population: Safety Analysis Set (all dosed participants). Methods: Descriptive statistics for categorical (TEAEs by MedDRA, severity) and continuous data (mean, median, SD, outliers); no inferential statistics or hypothesis testing (SAP Section 6.6).	Review: Clinical review by investigators and sponsors to identify dose-limiting toxicities; Safety Review Committee guides dose escalation based on unblinded data (SAP Section 6.6).	Prespecified endpoints, defined analysis set, suitable descriptive methods, bias mitigation via randomization; minimal robustness analyses acceptable for Phase 1 (ICH E9 Sections 2.3, 5.1, 5.5, 6.4).
S4	Endpoint: Incidence of adverse events (AEs) in ALS patients, assessing long-term safety and tolerability of reldesemtiv (SAP Section 8.2; Protocol Synopsis).	Population: Safety Analysis Set (all patients receiving ≥1 dose). Methods: Descriptive statistics for AEs (frequency, percentage by MedDRA SOC/PT, severity, relatedness, SAEs); no hypothesis testing; includes discontinuations, exposure, labs, vitals (SAP Section 8.2).	AE frequency, seriousness, and relatedness assessed; differences between early-start vs. delayed-start groups may indicate exposure effects; no inferential claims (SAP Section 8.2).	Prespecified AEs, standardized MedDRA coding, comprehensive descriptive summaries, appropriate safety set; lacks longitudinal trends, graphical summaries, recurrent AE analysis (ICH E9 Sections 5.2, 6.1–6.4).
S5	Endpoint: PK parameters (Cmax, AUClast, AUC0–inf) for 400 mg maribavir, comparing Japanese vs. non-Hispanic Caucasian subjects (SAP not specified).	Methods: ANOVA on log-transformed PK parameters for geometric mean ratios (GMRs) with 90% CIs; power model for dose proportionality in Japanese subjects (SAP not specified).	GMR and 90% CI assess ethnic PK differences; slope (β) ≈ 1 in power model indicates dose proportionality (SAP not specified).	Prespecified endpoints, standard ANOVA/power model, 90% CIs, covariate matching (age, sex, BMI); appropriate for Phase 1 exploratory

				trial (ICH E9 Sections 2.1.3, 2.2.2, 5.1, 5.5, 5.7).
B1	Endpoints: PFS and OS in advanced melanoma, comparing pembrolizumab vs. ipilimumab (SAP Section 3.5.3.1).	Methods: Stratified log-rank test, Kaplan-Meier curves, Cox model for HR (stratified by PD-L1 expression); hierarchical testing for multiplicity; interim analyses with Lan-DeMets O'Brien-Fleming boundaries (SAP Section 3.5.3.1).	Significant PFS/OS improvement indicates pembrolizumab benefit; HR and CIs assess effect size; exploratory subgroup analyses by PD-L1 status (SAP Section 3.5.3.1).	Prespecified endpoints, ITT analysis, multiplicity control, robust methods; needs detailed sensitivity analyses and cautious subgroup interpretation (ICH E9 Sections 2.3, 5.1, 5.2, 5.5, 5.6).
B2	Endpoint: Treated annualized bleed rate (ABR) in Hemophilia A patients without FVIII inhibitors, for emicizumab (1.5 mg/kg weekly, 3 mg/kg biweekly) vs. no prophylaxis (SAP Section 2.3.1).	Method: Negative binomial regression with treatment group covariate, bleed count as dependent variable, log of observation time offset, addressing overdispersion (SAP Section 2.3.1).	Rate ratios with 95% CIs and p-values; significant bleed rate reduction indicates emicizumab efficacy (SAP Section 2.3.1).	Prespecified endpoint, ITT analysis, suitable negative binomial model, stratified randomization minimizes bias, clinically relevant, robust with sensitivity analyses (ICH E9 Sections 2.3, 3.5, 5.1, 5.2, 5.3, 5.5, 5.7).
B3	Endpoints: ORR (CR/PR per RECIST 1.1, IRC-assessed) and OS (time to death, censored at last alive date) (SAP Section 3.5.3.1).	ORR: Cochran-Mantel-Haenszel test, 99% CI for odds ratio, stratified by prior paclitaxel, region. OS: Log-rank test, Cox model HR with adjusted CI, stratified; interim analysis with Lan-DeMets O'Brien-Fleming boundary ($\alpha = 0.04$ final, 0.016 interim) (SAP Section 3.5.3.1).	ORR significance at $\alpha = 0.01$, OS at $\alpha = 0.04$ (final) or 0.016 (interim) supports efficacy; HR, CIs guide effect size (SAP Section 3.5.3.1).	Prespecified endpoints, ITT, multiplicity control, robust tests; needs clearer missing data and sensitivity analysis plans for ORR (ICH E9 Sections 2.3, 4.5, 5.1, 5.3, 5.5, 5.6, 5.7).
B4	Endpoint: Proportion of TNFα inhibitor-naïve AS patients achieving ASAS20 response at Week 16 (SAP Section 2.3.1).	Population: Full Analysis Set (randomized, ≥1 dose, ≥1 post-baseline assessment). Method: Logistic regression (covariates: treatment, TNFα status, region); hierarchical testing for multiplicity; non-responder imputation, LOCF sensitivity analyses; two-sided $\alpha = 0.05$ (SAP Section 2.3.1).	Significant ASAS20 response rate increase in secukinumab (75 mg or 150 mg) vs. placebo indicates efficacy; hierarchical testing controls type I error (SAP Section 2.3.1).	Prespecified endpoint, ITT-based FAS, robust logistic regression, bias minimized via randomization/blinding, multiplicity controlled, sensitivity analyses ensure robustness (ICH E9 Sections 2.2.2, 2.3, 3.1, 5.1, 5.2, 5.3, 5.5, 5.6).

B5	FDA: Proportion achieving IGA 0/1 with ≥2-point reduction at Week 16. EMA: IGA 0/1 and EASI-75 (≥75% EASI improvement) at Week 16 (SAP not specified).	Test: Cochran-Mantel-Haenszel test, stratified by region, severity. Multiplicity: FDA: Hierarchical gatekeeping (α = 0.025); EMA: Intersection-union testing (α = 0.025). Missing Data: Non-responder imputation; sensitivity analyses planned (SAP not specified).	FDA: Significant IGA response supports dupilumab efficacy. EMA: Both IGA and EASI-75 significance required; hierarchical testing controls error (SAP not specified).	Prespecified endpoints, randomized design, robust CMH test, multiplicity control, conservative imputation; needs clearer covariate interactions, alternative estimators (ICH E9 Sections not specified).
G1	Endpoint: Early Bactericidal Activity (EBA), slope of $\log_{10}$ CFU/mL sputum change from baseline to Day 14, assessed in Stages 1 and 2 (SAP Sections 6.1.1, 8.1.1).	Methods: Two-time point EBA slope; linear mixed-effects model (fixed: time, treatment, interaction; random: subject intercepts); Fieller's and Taylor's (Delta) methods for EBA ratio CIs (SAP Sections 6.1.1, 8.1.1).	EBA slope indicates bactericidal activity; differences vs. RHEZ control show OPC-167832 efficacy; exposure-response modeling supports effect (SAP Sections 6.1.1, 8.1.1).	Prespecified EBA, robust mixed model, valid CI methods, appropriate longitudinal handling, clinically relevant, no multiplicity issues (ICH E9 Section 5).
G2	Endpoints: Maternal death/sepsis (42 days postpartum) and neonatal death/sepsis (28 days postpartum) (SAP not specified).	Populations: ITT (primary), High-Risk Cohort, As-Treated (sensitivity). Method: Generalized Linear Model (log link) for relative risk (RR) with 95% CIs, adjusted for site, robust standard errors for clustering; no multiplicity adjustment (SAP not specified).	Significant RR < 1 (95% CI excluding 1) supports azithromycin efficacy; independent evaluation of each endpoint (SAP not specified).	Prespecified endpoints, ITT analysis, randomization, robust GLM; partial concern for multiplicity without formal adjustment, needs clarification (ICH E9 Sections not specified).
G3	Endpoint: PCR-corrected Adequate Clinical and Parasitological Response (ACPR) at Day 29 in FAS (SAP Section 2.5.1).	Population: FAS (ITT). Method: Clopper-Pearson for 95% CI of ACPR proportion; Kaplan-Meier for supportive time-to-event; missing/indeterminate PCR data as failures. Sensitivity: Alternative PCR reclassification, non-study drug impact (SAP Section 2.5.1).	Dose arm effective if lower 95% CI bound > 80%, per WHO benchmarks (SAP Section 2.5.1).	Prespecified endpoint, ITT, conservative imputation, robust Clopper-Pearson, sensitivity analyses; needs 80% threshold justification, multiplicity plan for secondary endpoints (ICH E9 Sections 5.1, 5.2, 5.3, 5.5, 5.7).
G4	Endpoint: Days required for clinical improvement in mild to moderate	Primary: Kaplan-Meier, log-rank test, Cox model for HR with 95% CIs. Secondary: Logistic regression for odds ratios at ≤7	HR > 1 (95% CI excluding 1, 2-sided α = 0.05) indicates ivermectin +	Prespecified endpoint, appropriate Kaplan-Meier/Cox methods, ITT, randomization; lacks missing data

	COVID-19 patients (SAP Section 6.1).	and ≤12 days, adjusting for age, severity (SAP Sections 6.1.3, 6.1.4).	doxycycline superiority (SAP Section 6.1.3).	sensitivity analysis (ICH E9 Sections 2.2.2, 5.1, 5.3, 5.5, 5.7, 5.8).
G5	Endpoint: Vaccine Efficacy (VE) to prevent HIV-1 infection over 24 months in HIV-seronegative adults (SAP Section 7.1). Endpoints: AEs, SAEs, AESIs, reactogenicity (local/systemic within 3 days) (SAP Section 7.1).	Population: MITT (HIV-1 negative, ≥1 injection). Method: Nelson-Aalen for cumulative incidence, stratified VE by sex; Cox model (sex-stratified); TMLE, per-protocol, subgroup sensitivity analyses; one-sided test for VE = 0% (SAP Sections 4, 10.3.1, 10.3.2). Population: Safety Cohort (≥1 injection, as-treated). Method: Descriptive statistics (frequencies, proportions); Barnard's test for comparisons; no hypothesis testing (SAP Section 9).	Lower 95% CI bound > 0% indicates efficacy; futility boundary triggered early termination (SAP Sections 5, 11.2). Similar AE/SAE/AESI frequency/severity to placebo indicates safety; adverse trends trigger PSRT/DSMB review (SAP Sections 9, 11.1).	Prespecified endpoint, MITT, randomization, DSMB monitoring, robust methods; minor gap: lacks estimands per ICH E9(R1) (ICH E9 Sections 2.3, 3.1, 4.6, 5.1, 5.2, 5.3, 5.7). Prespecified endpoints, clear safety cohort, descriptive methods, Barnard's test, DSMB oversight; fully compliant (ICH E9 Sections 4.6, 5.2, 6.1–6.4).
